# P-875. Evaluation of the treatment of infections caused by Stenotrophomonas maltophilia

**DOI:** 10.1093/ofid/ofaf695.1083

**Published:** 2026-01-11

**Authors:** Carissa Hickey, Stephen May, Nicole Harrington, Caragh Clayton, Jessica Leri, Jennifer Wolf

**Affiliations:** Temple University Hospital, Lititz, PA; Christiana Care Health System, Newark, DE; ChristianaCare, Newark, Delaware; ChristianaCare, Newark, Delaware; Christiana Care Health System, Newark, DE; ChristianaCare, Newark, Delaware

## Abstract

**Background:**

The treatment of *Stenotrophomonas maltophilia* (*SMA*) presents many unique challenges. It is highly resistant and able to colonize medical lines and tubing. This study aimed to associate real-world practices with guideline-driven recommendations for the treatment of *SMA* infections.

**Methods:**

This descriptive retrospective chart review evaluated the initiation of antimicrobial therapy, either monotherapy, combination therapy, or not treated, in hospitalized adult patients at ChristianaCare within 48 hours of a positive *SMA* culture. The study compares prescribing practices pre- and post-publication of the Infectious Diseases Society of America (IDSA) guidelines on antimicrobial resistant gram-negative infections that recommended all *SMA* infections be treated with combination therapy. Patients were eligible for inclusion if they had a positive blood or respiratory culture between January 2020 to December 2021 or between January 2023 to July 2024. Exclusion criteria were death prior to culture speciation or transition to hospice within 30 days of positive culture. Outcomes were measured in percentages; no statistical tests were measured.

**Results:**

In the pre- and post- groups respectively, 17 of 50 patients (34%) compared to 15 of 50 patients (30%) were not treated, 32 of 50 patients (64%) versus 23 of 50 patients (46%) were treated with monotherapy, and 1 of 50 (2%) compared to 12 of 50 (24%) were started on combination therapy. There was a high incidence of polymicrobial cultures in the not treated and monotherapy groups. Overall Infectious Diseases consultations were frequent across all treatment categories but increased from the pre- to post- time periods. Furthermore, the use of levofloxacin in monotherapy decreased in the post- group (11%) compared to the pre- group (24%).

**Conclusion:**

The study found a small shift in *SMA* treatment practices at ChristianaCare towards combination therapy since the publication of IDSA guidelines. As a result, further education will be pursued to increased provider awareness of the recommended treatment strategies for *SMA* infections. In addition, investigators will continue to explore opportunities to use microbiology reports to encourage discernment between colonization and true infection.

**Disclosures:**

All Authors: No reported disclosures

